# Supplementary material for: Characterization of the Bacterial Profile from Natural and Laboratory Glossina Populations
Source: Insects. 2023 Oct 29;14(11):840. doi: 10.3390/insects14110840 (PMC10671886; doi:10.3390/insects14110840)
Supplement: Supplementary file 1 [file insects-14-00840-s001.zip › insects-2566057-supplementary-proof done.pdf]

Characterization of the bacterial profile from natural and laboratory *Glossina* populations

Youssef El Yamlahi[1,2,3], Naima Bel Mokhtar[1,3], Amal Maurady[1,2], Mohammed R. Britel[1], Costas Batargias[4], Delphina E. Mutembei[5], Hamis S. Nyingilili[5], Deusdedit J. Malulu[5], Imna I. Malele[6], Elias Asimakis[3], Panagiota Stathopoulou[3] and George Tsiamis [3*]

Supplementary Materials

Table S1: Representation and classification of OTUs identified in Glossina sp.

Feature ID	Read counts	% read counts	Phylum	Class	Order	Family	Genus
Otu1	2631218	39.6	Proteobacteria	Gammaproteobacteria	Enterobacteriales	Enterobacteriaceae	*Wigglesworthia*
Otu2	1071150	16.1	Proteobacteria	Gammaproteobacteria	Enterobacteriales	Enterobacteriaceae	*Sodalis*
Otu3	993528	14.9	Proteobacteria	Gammaproteobacteria	Enterobacteriales	Enterobacteriaceae	*Wigglesworthia*
Otu4	168767	2.5	Proteobacteria	Alphaproteobacteria	Rickettsiales	Anaplasmataceae	*Wolbachia*
Otu5	117636	1.8	Proteobacteria	Gammaproteobacteria	Pseudomonadales	Moraxellaceae	*Acinetobacter*
Otu6	67785	1.0	Proteobacteria	Alphaproteobacteria	Rhizobiales	Rhizobiaceae	*Phyllobacterium*
Otu7	61261	0.9	Firmicutes	Bacilli	Lactobacillales	Enterococcaceae	*Enterococcus*
Otu8	140733	2.1	Proteobacteria	Gammaproteobacteria	Enterobacteriales	Enterobacteriaceae	*Pseudocitrobacter*
Otu9	76803	1.2	Proteobacteria	Gammaproteobacteria	Pseudomonadales	Pseudomonadaceae	*Pseudomonas*
Otu10	47853	0.7	Proteobacteria	Gammaproteobacteria	Pseudomonadales	Moraxellaceae	*Psychrobacter*
Otu11	24118	0.4	Firmicutes	Bacilli	Bacillales	Planococcaceae	*Lysinibacillus*
Otu12	19936	0.3	Firmicutes	Bacilli	Bacillales	Bacillaceae	*Bacillus*
Otu13	22696	0.3	Proteobacteria	Gammaproteobacteria	Enterobacteriales	Enterobacteriaceae	*Enterobacter*
Otu14	12208	0.2	Proteobacteria	Gammaproteobacteria	Pseudomonadales	Moraxellaceae	*Acinetobacter*
Otu15	7127	0.1	Actinobacteria	Actinobacteria	Propionibacteriales	Propionibacteriaceae	*Cutibacterium*
Otu16	8993	0.1	Actinobacteria	Actinobacteria	Micrococcales	Micrococcaceae	*Kocuria*
Otu17	7447	0.1	Firmicutes	Bacilli	Bacillales	Family XII	*Exiguobacterium*
Otu19	7302	0.1	Proteobacteria	Alphaproteobacteria	Sphingomonadales	Sphingomonadaceae	*Novosphingobium*
Otu23	6396	0.1	Proteobacteria	Alphaproteobacteria	Sphingomonadales	Sphingomonadaceae	*Sphingomonas*
Otu28	7102	0.1	Proteobacteria	Gammaproteobacteria	Pseudomonadales	Pseudomonadaceae	*Pseudomonas*
Otu31	6072	0.1	Firmicutes	Bacilli	Bacillales	Staphylococcaceae	*Staphylococcus*
Otu34	3806	0.1	Firmicutes	Bacilli	Bacillales	Planococcaceae	*Planomicrobium*
Otu35	5346	0.1	Actinobacteria	Actinobacteria	Micrococcales	Micrococcaceae	*Arthrobacter*
Otu43	59368	0.9	Proteobacteria	Gammaproteobacteria	Enterobacteriales	Enterobacteriaceae	*Wigglesworthia*
Otu50	82835	1.2	Proteobacteria	Gammaproteobacteria	Enterobacteriales	Enterobacteriaceae	Sodalis
Otu58	935857	14.1	Proteobacteria	Gammaproteobacteria	Enterobacteriales	Enterobacteriaceae	Wigglesworthia
Otu74	11447	0.2	Proteobacteria	Gammaproteobacteria	Pseudomonadales	Moraxellaceae	Acinetobacter
Otu84	35938	0.5	Proteobacteria	Gammaproteobacteria	Enterobacteriales	Enterobacteriaceae	Wigglesworthia
Otu86	8467	0.1	Proteobacteria	Gammaproteobacteria	Enterobacteriales	Enterobacteriaceae	Pantoea

Table S2: The relative abundance of the laboratory populations according to the genus level

Bacteria	*Glossina austeni*	*Glossina morsitans*	*Glossina pallidipes*
Acinetobacter	0.009±0.01	0±0	0±0
Sphingomonas	0.012±0.01	0.011±0.01	0.018±0.01
Novosphingobium	0.006±0	0.001±0	0.047±0.04
Enterococcus	0±0	0.056±0.06	0±0
Staphylococcus	0.254±0.2	0.137±0.07	0.035±0.02
Cutibacterium	0.152±0.1	0.285±0.18	0.172±0.13
Pantoea	0.05±0.03	0.134±0.1	0.975±0.91
Enterobacter	0.1±0.07	0.333±0.27	0.766±0.66
Bacillus	0.024±0.02	0.025±0.02	1.513±1.35
Lysinibacillus	0.822±0.45	0.041±0.02	1.272±1.1
Pseudomonas	1.12±0.73	1.358±0.87	0.158±0.11
Psychrobacter	1.987±1.24	1.617±0.97	0.215±0.13
Phyllobacterium	3.002±1.85	3.071±1.96	0.476±0.33
Sodalis	7.675±3.2	10.111±1.91	11.477±2.28
Wigglesworthia	84.786±5.54	82.821±4.36	82.875±3.54

Table S3: Interactions between bacterial taxa at the OTU level. Ga: *Glossina austeni*; Gm: *Glossina morsitans*; Gp: *Glossina pallidipes*; Gs: *Glossina swynnertoni*.

#Interaction	Samples	Interacting OTU1	Interacting OTU2	Partner 1	Partner 2	Type of interaction
1	Ga-Lab	Otu2	Otu11	*Sodalis*	*Lysinibacillus*	copresence
2	Ga-Lab	Otu84	Otu11	*Wigglesworthia*	*Lysinibacillus*	copresence
3	Ga-Lab	Otu84	Otu23	*Wigglesworthia*	*Sphingomonas*	mutual exclusion
4	Ga-Lab	Otu84	Otu43	*Wigglesworthia*	*Wigglesworthia*	copresence
5	Ga-Lab	Otu84	Otu58	*Wigglesworthia*	*Wigglesworthia*	mutual exclusion
6	Ga-Lab	Otu50	Otu11	*Sodalis*	*Lysinibacillus*	copresence
7	Ga-Lab	Otu50	Otu58	*Sodalis*	*Wigglesworthia*	mutual exclusion
8	Ga-Lab	Otu19	Otu58	*Novosphingobium*	*Wigglesworthia*	mutual exclusion
9	Ga-Lab	Otu19	Otu1	*Novosphingobium*	*Wigglesworthia*	copresence
10	Ga-Lab	Otu28	Otu58	*Pseudomonas*	*Wigglesworthia*	mutual exclusion
11	Ga-Lab	Otu43	Otu11	*Wigglesworthia*	*Lysinibacillus*	copresence
12	Ga-Lab	Otu23	Otu1	*Sphingomonas*	*Wigglesworthia*	copresence
13	Ga-Lab	Otu23	Otu58	*Sphingomonas*	*Wigglesworthia*	mutual exclusion
14	Ga-Lab	Otu1	Otu31	*Wigglesworthia*	*Staphylococcus*	copresence
15	Ga-Lab	Otu1	Otu86	*Wigglesworthia*	*Pantoea*	copresence

16	Ga-Lab	Otu6	Otu58	*Phyllobacterium*	*Wigglesworthia*	mutual exclusion
17	Ga-Lab	Otu13	Otu43	*Enterobacter*	*Wigglesworthia*	copresence
18	Ga-Lab	Otu13	Otu58	*Enterobacter*	*Wigglesworthia*	mutual exclusion
19	Ga-Lab	Otu15	Otu43	*Cutibacterium*	*Wigglesworthia*	copresence
20	Ga-Lab	Otu9	Otu43	*Pseudomonas*	*Wigglesworthia*	copresence
21	Ga-Lab	Otu9	Otu58	*Pseudomonas*	*Wigglesworthia*	mutual exclusion
22	Ga-Lab	Otu58	Otu2	*Wigglesworthia*	*Sodalis*	mutual exclusion
23	Ga-Lab	Otu58	Otu10	*Wigglesworthia*	*Psychrobacter*	mutual exclusion
24	Ga-Lab	Otu58	Otu11	*Wigglesworthia*	*Lysinibacillus*	mutual exclusion
25	Ga-Lab	Otu58	Otu15	*Wigglesworthia*	*Cutibacterium*	mutual exclusion
26	Ga-Lab	Otu58	Otu31	*Wigglesworthia*	*Staphylococcus*	mutual exclusion
27	Ga-Lab	Otu58	Otu43	*Wigglesworthia*	*Wigglesworthia*	mutual exclusion
28	Ga-Lab	Otu58	Otu86	*Wigglesworthia*	*Pantoea*	mutual exclusion
29	Gm-Doma	Otu13	Otu2	*Enterobacter*	*Sodalis*	mutual exclusion
30	Gm-Doma	Otu13	Otu86	*Enterobacter*	*Pantoea*	copresence
31	Gm-Doma	Otu8	Otu74	*Pseudocitrobacter*	*Acinetobacter*	mutual exclusion
32	Gm-Doma	Otu74	Otu43	*Acinetobacter*	*Wigglesworthia*	mutual exclusion
33	Gm-Doma	Otu7	Otu3	*Enterococcus*	*Wigglesworthia*	mutual exclusion
34	Gm-Doma	Otu7	Otu74	*Enterococcus*	*Acinetobacter*	mutual exclusion
35	Gm-Doma	Otu1	Otu2	*Wigglesworthia*	*Sodalis*	mutual exclusion
36	Gm-Doma	Otu1	Otu43	*Wigglesworthia*	*Wigglesworthia*	mutual exclusion
37	Gm-Doma	Otu12	Otu74	*Bacillus*	*Acinetobacter*	mutual exclusion
38	Gm-Doma	Otu12	Otu13	*Bacillus*	*Enterobacter*	mutual exclusion
39	Gm-Doma	Otu12	Otu31	*Bacillus*	*Staphylococcus*	copresence
40	Gm-Doma	Otu15	Otu31	*Cutibacterium*	*Staphylococcus*	copresence
41	Gm-Doma	Otu15	Otu86	*Cutibacterium*	*Pantoea*	copresence
42	Gm-Doma	Otu86	Otu31	*Pantoea*	*Staphylococcus*	copresence
43	Gm-Doma	Otu84	Otu15	*Wigglesworthia*	*Cutibacterium*	copresence
44	Gm-Doma	Otu84	Otu31	*Wigglesworthia*	*Staphylococcus*	copresence
45	Gm-Doma	Otu84	Otu43	*Wigglesworthia*	*Wigglesworthia*	copresence
46	Gm-Doma	Otu84	Otu86	*Wigglesworthia*	*Pantoea*	copresence
47	Gm-Doma	Otu28	Otu2	*Pseudomonas*	*Sodalis*	mutual exclusion
48	Gm-Doma	Otu50	Otu74	*Sodalis*	*Acinetobacter*	mutual exclusion
49	Gm-Doma	Otu50	Otu84	*Sodalis*	*Wigglesworthia*	copresence
50	Gm-Doma	Otu50	Otu1	*Sodalis*	*Wigglesworthia*	mutual exclusion

51	Gm-Doma	Otu50	Otu2	*Sodalis*	*Sodalis*	copresence
52	Gm-Doma	Otu19	Otu12	*Novosphingobium*	*Bacillus*	copresence
53	Gm-Doma	Otu19	Otu15	*Novosphingobium*	*Cutibacterium*	copresence
54	Gm-Doma	Otu19	Otu31	*Novosphingobium*	*Staphylococcus*	copresence
55	Gm-Doma	Otu2	Otu43	*Sodalis*	*Wigglesworthia*	copresence
56	Gm-Doma	Otu2	Otu74	*Sodalis*	*Acinetobacter*	mutual exclusion
57	Gm-Doma	Otu23	Otu2	*Sphingomonas*	*Sodalis*	mutual exclusion
58	Gm-Doma	Otu3	Otu86	*Wigglesworthia*	*Pantoea*	copresence
59	Gm-Doma	Otu3	Otu2	*Wigglesworthia*	*Sodalis*	mutual exclusion
60	Gm-Doma	Otu3	Otu4	*Wigglesworthia*	*Wolbachia*	copresence
61	Gm-Doma	Otu4	Otu1	*Wolbachia*	*Wigglesworthia*	mutual exclusion
62	Gm-Doma	Otu4	Otu2	*Wolbachia*	*Sodalis*	mutual exclusion
63	Gm-Doma	Otu4	Otu23	*Wolbachia*	*Sphingomonas*	copresence
64	Gm-Doma	Otu4	Otu28	*Wolbachia*	*Pseudomonas*	copresence
65	Gm-Doma	Otu4	Otu31	*Wolbachia*	*Staphylococcus*	copresence
66	Gm-Doma	Otu4	Otu86	*Wolbachia*	*Pantoea*	copresence
67	Gm-Lab	Otu9	Otu3	*Pseudomonas*	*Wigglesworthia*	mutual exclusion
68	Gm-Lab	Otu9	Otu11	*Pseudomonas*	*Lysinibacillus*	copresence
69	Gm-Lab	Otu9	Otu43	*Pseudomonas*	*Wigglesworthia*	mutual exclusion
70	Gm-Lab	Otu9	Otu50	*Pseudomonas*	*Sodalis*	mutual exclusion
71	Gm-Lab	Otu2	Otu43	*Sodalis*	*Wigglesworthia*	copresence
72	Gm-Lab	Otu19	Otu23	*Novosphingobium*	*Sphingomonas*	copresence
73	Gm-Lab	Otu84	Otu13	*Wigglesworthia*	*Enterobacter*	copresence
74	Gm-Lab	Otu84	Otu15	*Wigglesworthia*	*Cutibacterium*	copresence
75	Gm-Lab	Otu84	Otu28	*Wigglesworthia*	*Pseudomonas*	copresence
76	Gm-Lab	Otu84	Otu31	*Wigglesworthia*	*Staphylococcus*	copresence
77	Gm-Lab	Otu84	Otu1	*Wigglesworthia*	*Wigglesworthia*	copresence
78	Gm-Lab	Otu15	Otu43	*Cutibacterium*	*Wigglesworthia*	mutual exclusion
79	Gm-Lab	Otu28	Otu23	*Pseudomonas*	*Sphingomonas*	copresence
80	Gm-Lab	Otu6	Otu11	*Phyllobacterium*	*Lysinibacillus*	copresence
81	Gm-Lab	Otu6	Otu43	*Phyllobacterium*	*Wigglesworthia*	mutual exclusion
82	Gm-Lab	Otu31	Otu43	*Staphylococcus*	*Wigglesworthia*	mutual exclusion
83	Gm-Lab	Otu50	Otu10	*Sodalis*	*Psychrobacter*	mutual exclusion
84	Gm-Lab	Otu50	Otu23	*Sodalis*	*Sphingomonas*	mutual exclusion
85	Gm-Lab	Otu50	Otu2	*Sodalis*	*Sodalis*	copresence
86	Gm-Lab	Otu50	Otu6	*Sodalis*	*Phyllobacterium*	mutual exclusion
87	Gm-Lab	Otu43	Otu10	*Wigglesworthia*	*Psychrobacter*	mutual exclusion
88	Gm-Lab	Otu43	Otu11	*Wigglesworthia*	*Lysinibacillus*	mutual exclusion
89	Gm-Lab	Otu12	Otu43	*Bacillus*	*Wigglesworthia*	mutual exclusion

90	Gm-Lab	Otu13	Otu43	*Enterobacter*	*Wigglesworthia*	mutual exclusion
91	Gm-Lab	Otu23	Otu10	*Sphingomonas*	*Psychrobacter*	copresence
92	Gm-Lab	Otu23	Otu11	*Sphingomonas*	*Lysinibacillus*	copresence
93	Gm-Lab	Otu23	Otu31	*Sphingomonas*	*Staphylococcus*	copresence
94	Gm-Lab	Otu23	Otu43	*Sphingomonas*	*Wigglesworthia*	mutual exclusion
95	Gm-Lab	Otu3	Otu1	*Wigglesworthia*	*Wigglesworthia*	mutual exclusion
96	Gm-Lab	Otu3	Otu2	*Wigglesworthia*	*Sodalis*	mutual exclusion
97	Gm-Lab	Otu3	Otu6	*Wigglesworthia*	*Phyllobacterium*	mutual exclusion
98	Gm-Lab	Otu3	Otu10	*Wigglesworthia*	*Psychrobacter*	mutual exclusion
99	Gm-Lab	Otu3	Otu11	*Wigglesworthia*	*Lysinibacillus*	mutual exclusion
100	Gm-Lab	Otu3	Otu12	*Wigglesworthia*	*Bacillus*	mutual exclusion
101	Gm-Lab	Otu3	Otu13	*Wigglesworthia*	*Enterobacter*	mutual exclusion
102	Gm-Lab	Otu3	Otu15	*Wigglesworthia*	*Cutibacterium*	mutual exclusion
103	Gm-Lab	Otu3	Otu23	*Wigglesworthia*	*Sphingomonas*	mutual exclusion
104	Gm-Lab	Otu3	Otu28	*Wigglesworthia*	*Pseudomonas*	mutual exclusion
105	Gm-Lab	Otu3	Otu31	*Wigglesworthia*	*Staphylococcus*	mutual exclusion
106	Gm-Lab	Otu3	Otu58	*Wigglesworthia*	*Wigglesworthia*	mutual exclusion
107	Gm-Lab	Otu3	Otu86	*Wigglesworthia*	*Pantoea*	mutual exclusion
108	Gm-Lab	Otu58	Otu1	*Wigglesworthia*	*Wigglesworthia*	copresence
109	Gm-Lab	Otu58	Otu11	*Wigglesworthia*	*Lysinibacillus*	copresence
110	Gm-Lab	Otu58	Otu43	*Wigglesworthia*	*Wigglesworthia*	mutual exclusion
111	Gp-Doma	Otu84	Otu23	*Wigglesworthia*	*Sphingomonas*	mutual exclusion
112	Gp-Doma	Otu84	Otu28	*Wigglesworthia*	*Pseudomonas*	mutual exclusion
113	Gp-Doma	Otu84	Otu2	*Wigglesworthia*	*Sodalis*	copresence
114	Gp-Doma	Otu74	Otu10	*Acinetobacter*	*Psychrobacter*	mutual exclusion
115	Gp-Doma	Otu7	Otu3	*Enterococcus*	*Wigglesworthia*	mutual exclusion
116	Gp-Doma	Otu7	Otu13	*Enterococcus*	*Enterobacter*	mutual exclusion
117	Gp-Doma	Otu7	Otu43	*Enterococcus*	*Wigglesworthia*	mutual exclusion
118	Gp-Doma	Otu7	Otu58	*Enterococcus*	*Wigglesworthia*	mutual exclusion
119	Gp-Doma	Otu23	Otu2	*Sphingomonas*	*Sodalis*	mutual exclusion
120	Gp-Doma	Otu28	Otu43	*Pseudomonas*	*Wigglesworthia*	mutual exclusion
121	Gp-Doma	Otu8	Otu3	*Pseudocitrobacter*	*Wigglesworthia*	mutual exclusion

122	Gp-Doma	Otu8	Otu12	*Pseudocitrobacter*	*Bacillus*	mutual exclusion
123	Gp-Doma	Otu8	Otu13	*Pseudocitrobacter*	*Enterobacter*	mutual exclusion
124	Gp-Doma	Otu8	Otu43	*Pseudocitrobacter*	*Wigglesworthia*	mutual exclusion
125	Gp-Doma	Otu8	Otu50	*Pseudocitrobacter*	*Sodalis*	mutual exclusion
126	Gp-Doma	Otu8	Otu58	*Pseudocitrobacter*	*Wigglesworthia*	mutual exclusion
127	Gp-Doma	Otu9	Otu12	*Pseudomonas*	*Bacillus*	mutual exclusion
128	Gp-Doma	Otu9	Otu15	*Pseudomonas*	*Cutibacterium*	mutual exclusion
129	Gp-Doma	Otu9	Otu43	*Pseudomonas*	*Wigglesworthia*	mutual exclusion
130	Gp-Doma	Otu9	Otu5	*Pseudomonas*	*Acinetobacter*	mutual exclusion
131	Gp-Doma	Otu9	Otu74	*Pseudomonas*	*Acinetobacter*	mutual exclusion
132	Gp-Doma	Otu86	Otu31	*Pantoea*	*Staphylococcus*	copresence
133	Gp-Doma	Otu2	Otu43	*Sodalis*	*Wigglesworthia*	copresence
134	Gp-Doma	Otu58	Otu10	*Wigglesworthia*	*Psychrobacter*	mutual exclusion
135	Gp-Doma	Otu58	Otu86	*Wigglesworthia*	*Pantoea*	copresence
136	Gp-Doma	Otu43	Otu10	*Wigglesworthia*	*Psychrobacter*	mutual exclusion
137	Gp-Doma	Otu15	Otu10	*Cutibacterium*	*Psychrobacter*	mutual exclusion
138	Gp-Doma	Otu15	Otu31	*Cutibacterium*	*Staphylococcus*	copresence
139	Gp-Doma	Otu31	Otu11	*Staphylococcus*	*Lysinibacillus*	copresence
140	Gp-Doma	Otu19	Otu31	*Novosphingobium*	*Staphylococcus*	copresence
141	Gp-Doma	Otu3	Otu86	*Wigglesworthia*	*Pantoea*	copresence
142	Gp-Doma	Otu3	Otu10	*Wigglesworthia*	*Psychrobacter*	mutual exclusion
143	Gp-Doma	Otu3	Otu5	*Wigglesworthia*	*Acinetobacter*	mutual exclusion
144	Gp-Doma	Otu3	Otu6	*Wigglesworthia*	*Phyllobacterium*	mutual exclusion
145	Gp-Doma	Otu50	Otu2	*Sodalis*	*Sodalis*	copresence
146	Gp-Doma	Otu50	Otu23	*Sodalis*	*Sphingomonas*	mutual exclusion
147	Gp-Doma	Otu50	Otu28	*Sodalis*	*Pseudomonas*	mutual exclusion
148	Gp-Doma	Otu10	Otu11	*Psychrobacter*	*Lysinibacillus*	mutual exclusion
149	Gp-Doma	Otu6	Otu11	*Phyllobacterium*	*Lysinibacillus*	mutual exclusion
150	Gp-Doma	Otu6	Otu12	*Phyllobacterium*	*Bacillus*	mutual exclusion
151	Gp-Doma	Otu6	Otu13	*Phyllobacterium*	*Enterobacter*	mutual exclusion
152	Gp-Doma	Otu6	Otu15	*Phyllobacterium*	*Cutibacterium*	mutual exclusion
153	Gp-Doma	Otu6	Otu58	*Phyllobacterium*	*Wigglesworthia*	mutual exclusion

154	Gp-Doma	Otu6	Otu74	*Phyllobacterium*	*Acinetobacter*	mutual exclusion
155	Gp-Doma	Otu6	Otu86	*Phyllobacterium*	*Pantoea*	mutual exclusion
156	Gp-Doma	Otu13	Otu10	*Enterobacter*	*Psychrobacter*	mutual exclusion
157	Gp-Doma	Otu12	Otu10	*Bacillus*	*Psychrobacter*	mutual exclusion
158	Gp-Doma	Otu12	Otu15	*Bacillus*	*Cutibacterium*	copresence
159	Gp-Doma	Otu12	Otu31	*Bacillus*	*Staphylococcus*	copresence
160	Gp-Doma	Otu5	Otu19	*Acinetobacter*	*Novosphingobium*	mutual exclusion
161	Gp-Doma	Otu5	Otu31	*Acinetobacter*	*Staphylococcus*	mutual exclusion
162	Gp-Doma	Otu5	Otu86	*Acinetobacter*	*Pantoea*	mutual exclusion
163	Gp-Doma	Otu5	Otu6	*Acinetobacter*	*Phyllobacterium*	mutual exclusion
164	Gp-Doma	Otu5	Otu10	*Acinetobacter*	*Psychrobacter*	mutual exclusion
165	Gp-Doma	Otu5	Otu11	*Acinetobacter*	*Lysinibacillus*	mutual exclusion
166	Gp-Doma	Otu5	Otu12	*Acinetobacter*	*Bacillus*	mutual exclusion
167	Gp-Lab	Otu84	Otu1	*Wigglesworthia*	*Wigglesworthia*	mutual exclusion
168	Gp-Lab	Otu28	Otu10	*Pseudomonas*	*Psychrobacter*	copresence
169	Gp-Lab	Otu28	Otu19	*Pseudomonas*	*Novosphingobium*	copresence
170	Gp-Lab	Otu28	Otu23	*Pseudomonas*	*Sphingomonas*	copresence
171	Gp-Lab	Otu19	Otu23	*Novosphingobium*	*Sphingomonas*	copresence
172	Gp-Lab	Otu31	Otu10	*Staphylococcus*	*Psychrobacter*	copresence
173	Gp-Lab	Otu23	Otu15	*Sphingomonas*	*Cutibacterium*	copresence
174	Gp-Lab	Otu23	Otu31	*Sphingomonas*	*Staphylococcus*	copresence
175	Gp-Lab	Otu58	Otu1	*Wigglesworthia*	*Wigglesworthia*	mutual exclusion
176	Gp-Lab	Otu50	Otu1	*Sodalis*	*Wigglesworthia*	mutual exclusion
177	Gp-Lab	Otu13	Otu1	*Enterobacter*	*Wigglesworthia*	mutual exclusion
178	Gp-Lab	Otu1	Otu2	*Wigglesworthia*	*Sodalis*	mutual exclusion
179	Gp-Lab	Otu1	Otu15	*Wigglesworthia*	*Cutibacterium*	mutual exclusion
180	Gp-Lab	Otu1	Otu43	*Wigglesworthia*	*Wigglesworthia*	mutual exclusion
181	Gp-Lab	Otu3	Otu1	*Wigglesworthia*	*Wigglesworthia*	mutual exclusion
182	Gp-Lab	Otu6	Otu23	*Phyllobacterium*	*Sphingomonas*	copresence
183	Gp-Lab	Otu6	Otu31	*Phyllobacterium*	*Staphylococcus*	copresence
184	Gp-Makao	Otu84	Otu1	*Wigglesworthia*	*Wigglesworthia*	mutual exclusion
185	Gp-Makao	Otu86	Otu31	*Pantoea*	*Staphylococcus*	copresence
186	Gp-Makao	Otu16	Otu13	*Kocuria*	*Enterobacter*	mutual exclusion
187	Gp-Makao	Otu16	Otu5	*Kocuria*	*Acinetobacter*	mutual exclusion

188	Gp-Makao	Otu16	Otu74	*Kocuria*	*Acinetobacter*	mutual exclusion
189	Gp-Makao	Otu1	Otu2	*Wigglesworthia*	*Sodalis*	mutual exclusion
190	Gp-Makao	Otu1	Otu43	*Wigglesworthia*	*Wigglesworthia*	mutual exclusion
191	Gp-Makao	Otu28	Otu19	*Pseudomonas*	*Novosphingobium*	copresence
192	Gp-Makao	Otu28	Otu86	*Pseudomonas*	*Pantoea*	copresence
193	Gp-Makao	Otu5	Otu14	*Acinetobacter*	*Acinetobacter*	mutual exclusion
194	Gp-Makao	Otu5	Otu34	*Acinetobacter*	*Planomicrobium*	mutual exclusion
195	Gp-Makao	Otu5	Otu35	*Acinetobacter*	*Arthrobacter*	mutual exclusion
196	Gp-Makao	Otu5	Otu43	*Acinetobacter*	*Wigglesworthia*	mutual exclusion
197	Gp-Makao	Otu5	Otu2	*Acinetobacter*	*Sodalis*	mutual exclusion
198	Gp-Makao	Otu13	Otu14	*Enterobacter*	*Acinetobacter*	mutual exclusion
199	Gp-Makao	Otu13	Otu31	*Enterobacter*	*Staphylococcus*	copresence
200	Gp-Makao	Otu13	Otu35	*Enterobacter*	*Arthrobacter*	mutual exclusion
201	Gp-Makao	Otu13	Otu2	*Enterobacter*	*Sodalis*	mutual exclusion
202	Gp-Makao	Otu74	Otu43	*Acinetobacter*	*Wigglesworthia*	mutual exclusion
203	Gp-Makao	Otu17	Otu5	*Exiguobacterium*	*Acinetobacter*	mutual exclusion
204	Gp-Makao	Otu17	Otu13	*Exiguobacterium*	*Enterobacter*	mutual exclusion
205	Gp-Makao	Otu17	Otu74	*Exiguobacterium*	*Acinetobacter*	mutual exclusion
206	Gp-Makao	Otu2	Otu74	*Sodalis*	*Acinetobacter*	mutual exclusion
207	Gp-Makao	Otu50	Otu74	*Sodalis*	*Acinetobacter*	mutual exclusion
208	Gp-Makao	Otu50	Otu1	*Sodalis*	*Wigglesworthia*	mutual exclusion
209	Gp-Makao	Otu50	Otu5	*Sodalis*	*Acinetobacter*	mutual exclusion
210	Gp-Makao	Otu34	Otu13	*Planomicrobium*	*Enterobacter*	mutual exclusion
211	Gp-Makao	Otu35	Otu74	*Arthrobacter*	*Acinetobacter*	mutual exclusion
212	Gp-Makao	Otu19	Otu86	*Novosphingobium*	*Pantoea*	copresence
213	Gp-Makao	Otu19	Otu23	*Novosphingobium*	*Sphingomonas*	copresence
214	Gp-Makao	Otu19	Otu31	*Novosphingobium*	*Staphylococcus*	copresence
215	Gp-Makao	Otu19	Otu34	*Novosphingobium*	*Planomicrobium*	copresence
216	Gp-Makao	Otu19	Otu35	*Novosphingobium*	*Arthrobacter*	copresence
217	Gp-Msubugwe	Otu2	Otu31	*Sodalis*	*Staphylococcus*	mutual exclusion
218	Gp-Msubugwe	Otu50	Otu1	*Sodalis*	*Wigglesworthia*	mutual exclusion
219	Gp-Msubugwe	Otu1	Otu43	*Wigglesworthia*	*Wigglesworthia*	mutual exclusion

220	Gp-Msubugwe	Otu19	Otu23	*Novosphingobium*	*Sphingomonas*	copresence
221	Gp-Msubugwe	Otu28	Otu19	*Pseudomonas*	*Novosphingobium*	copresence
222	Gp-Msubugwe	Otu28	Otu23	*Pseudomonas*	*Sphingomonas*	copresence
223	Gp-Msubugwe	Otu23	Otu31	*Sphingomonas*	*Staphylococcus*	copresence
224	Gp-Msubugwe	Otu23	Otu43	*Sphingomonas*	*Wigglesworthia*	mutual exclusion
225	Gp-Msubugwe	Otu23	Otu2	*Sphingomonas*	*Sodalis*	mutual exclusion
226	Gp-Msubugwe	Otu31	Otu43	*Staphylococcus*	*Wigglesworthia*	mutual exclusion
227	Gp-Msubugwe	Otu84	Otu1	*Wigglesworthia*	*Wigglesworthia*	mutual exclusion
228	Gp-Msubugwe	Otu84	Otu23	*Wigglesworthia*	*Sphingomonas*	mutual exclusion
229	Gp-Msubugwe	Otu84	Otu31	*Wigglesworthia*	*Staphylococcus*	mutual exclusion
230	Gs-Makao	Otu28	Otu1	*Pseudomonas*	*Wigglesworthia*	copresence
231	Gs-Makao	Otu28	Otu5	*Pseudomonas*	*Acinetobacter*	mutual exclusion
232	Gs-Makao	Otu28	Otu23	*Pseudomonas*	*Sphingomonas*	copresence
233	Gs-Makao	Otu28	Otu86	*Pseudomonas*	*Pantoea*	copresence
234	Gs-Makao	Otu14	Otu31	*Acinetobacter*	*Staphylococcus*	copresence
235	Gs-Makao	Otu14	Otu86	*Acinetobacter*	*Pantoea*	copresence
236	Gs-Makao	Otu15	Otu31	*Cutibacterium*	*Staphylococcus*	copresence
237	Gs-Makao	Otu50	Otu10	*Sodalis*	*Psychrobacter*	mutual exclusion
238	Gs-Makao	Otu50	Otu1	*Sodalis*	*Wigglesworthia*	copresence
239	Gs-Makao	Otu50	Otu5	*Sodalis*	*Acinetobacter*	mutual exclusion
240	Gs-Makao	Otu9	Otu2	*Pseudomonas*	*Sodalis*	mutual exclusion
241	Gs-Makao	Otu9	Otu3	*Pseudomonas*	*Wigglesworthia*	mutual exclusion
242	Gs-Makao	Otu9	Otu50	*Pseudomonas*	*Sodalis*	mutual exclusion
243	Gs-Makao	Otu74	Otu11	*Acinetobacter*	*Lysinibacillus*	mutual exclusion
244	Gs-Makao	Otu74	Otu43	*Acinetobacter*	*Wigglesworthia*	mutual exclusion
245	Gs-Makao	Otu2	Otu10	*Sodalis*	*Psychrobacter*	mutual exclusion
246	Gs-Makao	Otu2	Otu74	*Sodalis*	*Acinetobacter*	mutual exclusion
247	Gs-Makao	Otu19	Otu10	*Novosphingobium*	*Psychrobacter*	copresence
248	Gs-Makao	Otu19	Otu23	*Novosphingobium*	*Sphingomonas*	copresence
249	Gs-Makao	Otu19	Otu31	*Novosphingobium*	*Staphylococcus*	copresence
250	Gs-Makao	Otu19	Otu86	*Novosphingobium*	*Pantoea*	copresence
251	Gs-Makao	Otu19	Otu6	*Novosphingobium*	*Phyllobacterium*	copresence
252	Gs-Makao	Otu34	Otu23	*Planomicrobium*	*Sphingomonas*	copresence
253	Gs-Makao	Otu34	Otu31	*Planomicrobium*	*Staphylococcus*	copresence
254	Gs-Makao	Otu35	Otu31	*Arthrobacter*	*Staphylococcus*	copresence
255	Gs-Makao	Otu35	Otu58	*Arthrobacter*	*Wigglesworthia*	mutual exclusion

256	Gs-Makao	Otu35	Otu86	*Arthrobacter*	*Pantoea*	copresence
257	Gs-Makao	Otu6	Otu2	*Phyllobacterium*	*Sodalis*	mutual exclusion
258	Gs-Makao	Otu3	Otu2	*Wigglesworthia*	*Sodalis*	mutual exclusion
259	Gs-Makao	Otu3	Otu6	*Wigglesworthia*	*Phyllobacterium*	mutual exclusion
260	Gs-Makao	Otu3	Otu10	*Wigglesworthia*	*Psychrobacter*	mutual exclusion
261	Gs-Makao	Otu3	Otu14	*Wigglesworthia*	*Acinetobacter*	mutual exclusion
262	Gs-Makao	Otu3	Otu16	*Wigglesworthia*	*Kocuria*	mutual exclusion
263	Gs-Makao	Otu3	Otu17	*Wigglesworthia*	*Exiguobacterium*	mutual exclusion
264	Gs-Makao	Otu3	Otu34	*Wigglesworthia*	*Planomicrobium*	mutual exclusion
265	Gs-Makao	Otu3	Otu35	*Wigglesworthia*	*Arthrobacter*	mutual exclusion
266	Gs-Makao	Otu84	Otu15	*Wigglesworthia*	*Cutibacterium*	copresence
267	Gs-Makao	Otu84	Otu23	*Wigglesworthia*	*Sphingomonas*	copresence
268	Gs-Makao	Otu84	Otu28	*Wigglesworthia*	*Pseudomonas*	copresence
269	Gs-Makao	Otu84	Otu31	*Wigglesworthia*	*Staphylococcus*	copresence
270	Gs-Makao	Otu84	Otu86	*Wigglesworthia*	*Pantoea*	copresence
271	Gs-Makao	Otu84	Otu5	*Wigglesworthia*	*Acinetobacter*	mutual exclusion
272	Gs-Makao	Otu1	Otu31	*Wigglesworthia*	*Staphylococcus*	copresence
273	Gs-Makao	Otu1	Otu43	*Wigglesworthia*	*Wigglesworthia*	copresence
274	Gs-Makao	Otu23	Otu14	*Sphingomonas*	*Acinetobacter*	copresence
275	Gs-Makao	Otu23	Otu15	*Sphingomonas*	*Cutibacterium*	copresence
276	Gs-Makao	Otu23	Otu35	*Sphingomonas*	*Arthrobacter*	copresence
277	Gs-Makao	Otu5	Otu1	*Acinetobacter*	*Wigglesworthia*	mutual exclusion
278	Gs-Makao	Otu5	Otu11	*Acinetobacter*	*Lysinibacillus*	mutual exclusion
279	Gs-Makao	Otu5	Otu13	*Acinetobacter*	*Enterobacter*	copresence
280	Gs-Makao	Otu5	Otu19	*Acinetobacter*	*Novosphingobium*	mutual exclusion
281	Gs-Makao	Otu5	Otu23	*Acinetobacter*	*Sphingomonas*	mutual exclusion
282	Gs-Makao	Otu5	Otu31	*Acinetobacter*	*Staphylococcus*	mutual exclusion
283	Gs-Makao	Otu5	Otu43	*Acinetobacter*	*Wigglesworthia*	mutual exclusion
284	Gs-Makao	Otu5	Otu58	*Acinetobacter*	*Wigglesworthia*	mutual exclusion
285	Gs-Makao	Otu5	Otu86	*Acinetobacter*	*Pantoea*	mutual exclusion
286	Gs-Makao	Otu58	Otu10	*Wigglesworthia*	*Psychrobacter*	mutual exclusion
287	Gs-Makao	Otu58	Otu74	*Wigglesworthia*	*Acinetobacter*	mutual exclusion

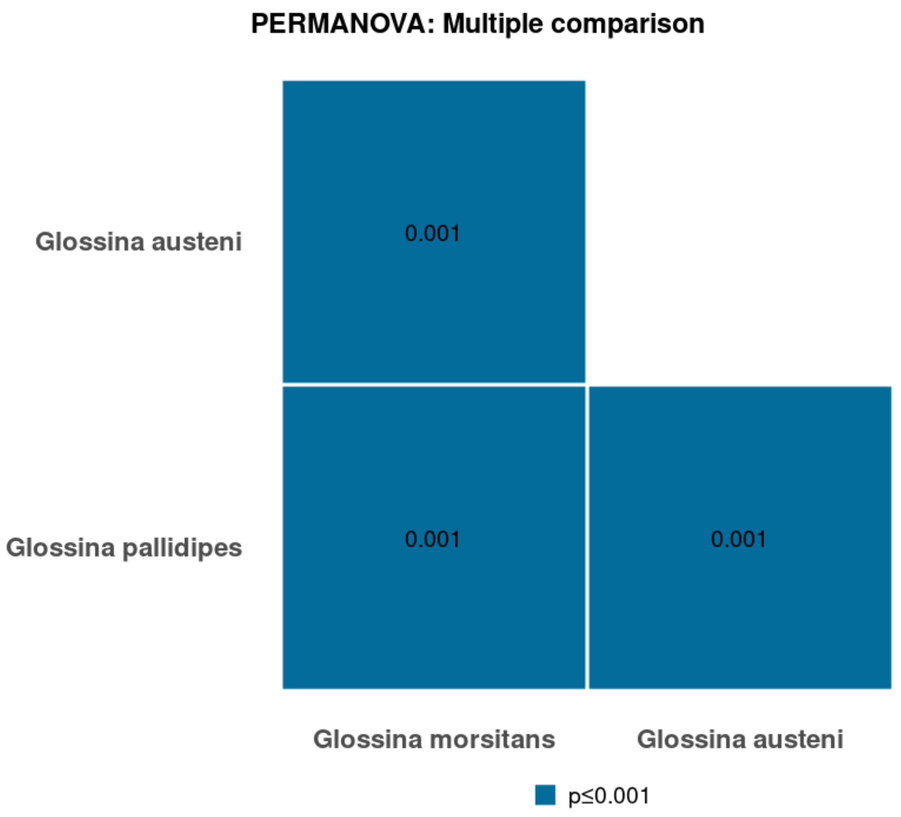

Figure S1: Beta diversity (pairwise comparison) of bacterial communities of lab samples based on fly species.

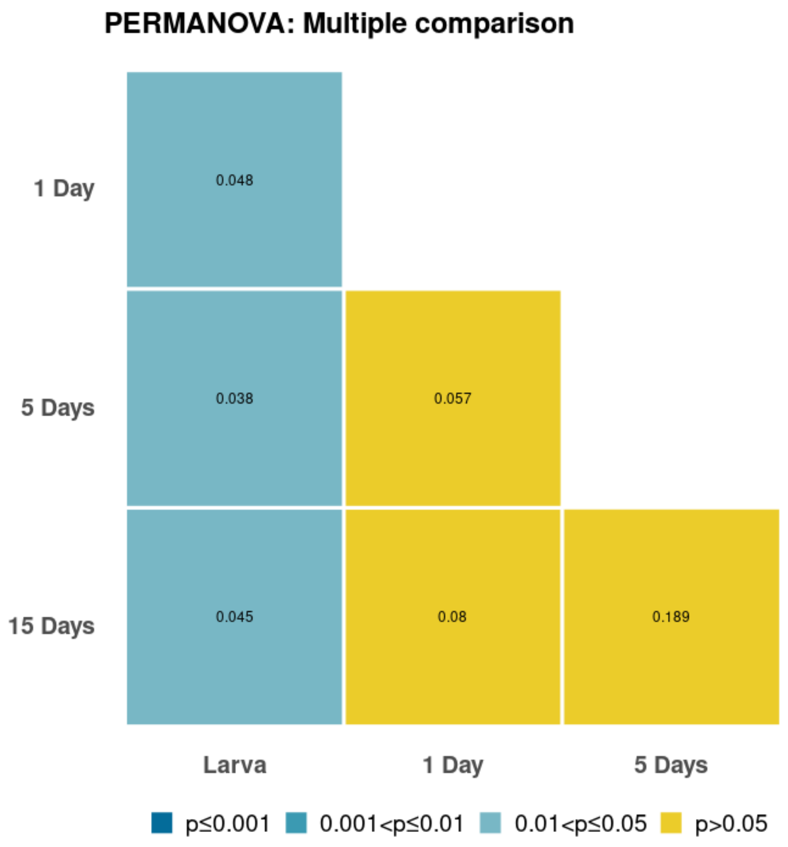

Figure S2: Summary of beta diversity results (pairwise comparison) of the laboratory samples of *G. austeni*

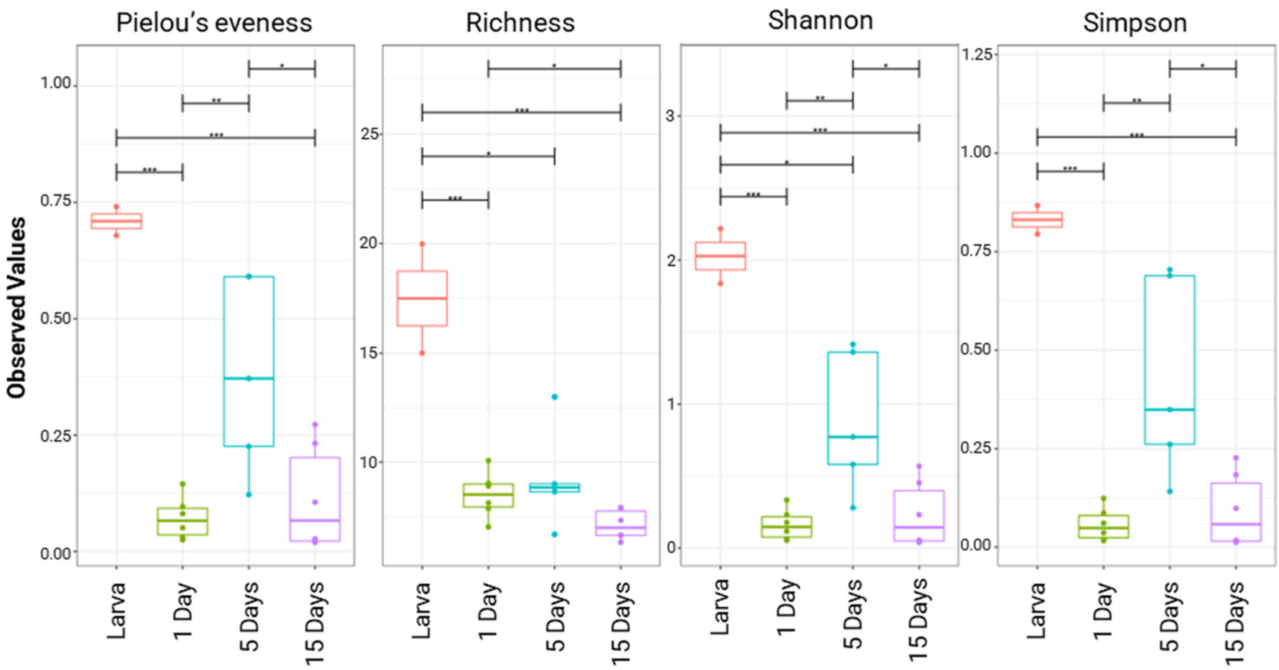

Figure S3: Species richness and diversity indices with significant differences of *G. austeni* samples according to age. Boxes represent inter-quartile range (IQR), the line within the boxes is the median, and the dots represent samples. * p < 0.05, ** p < 0.01 and *** p < 0.001.

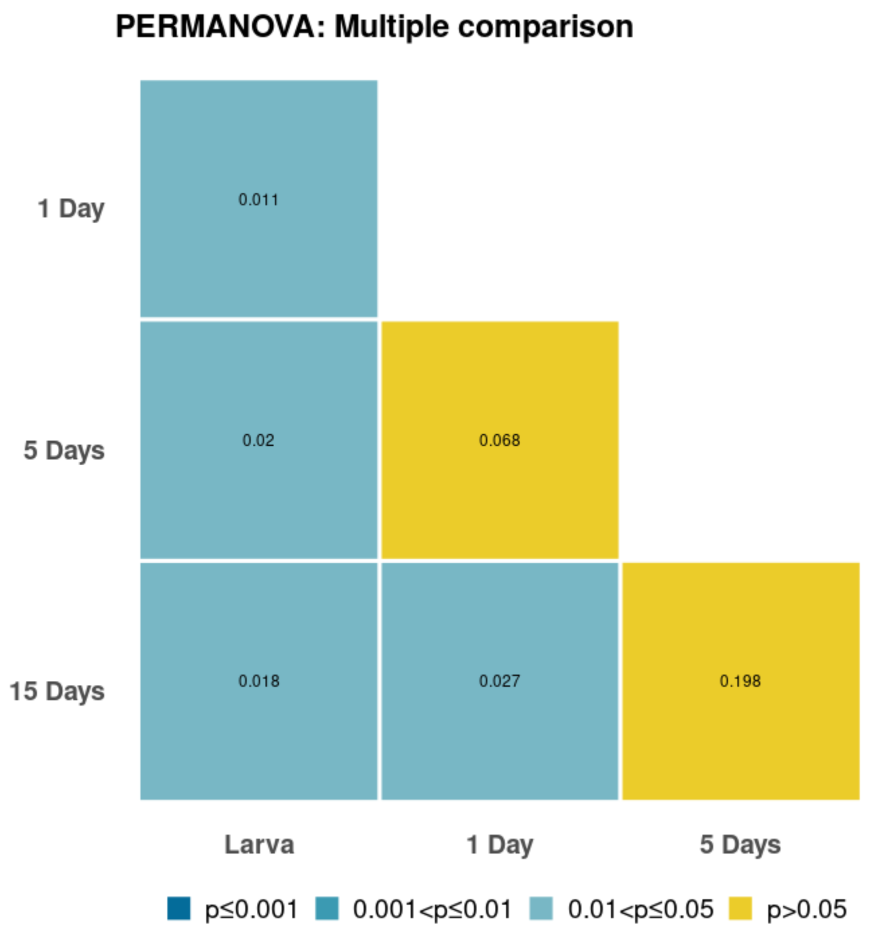

Figure S4: Summary of beta diversity results (pairwise comparison) of the laboratory samples of *G. morsitans morsitans*

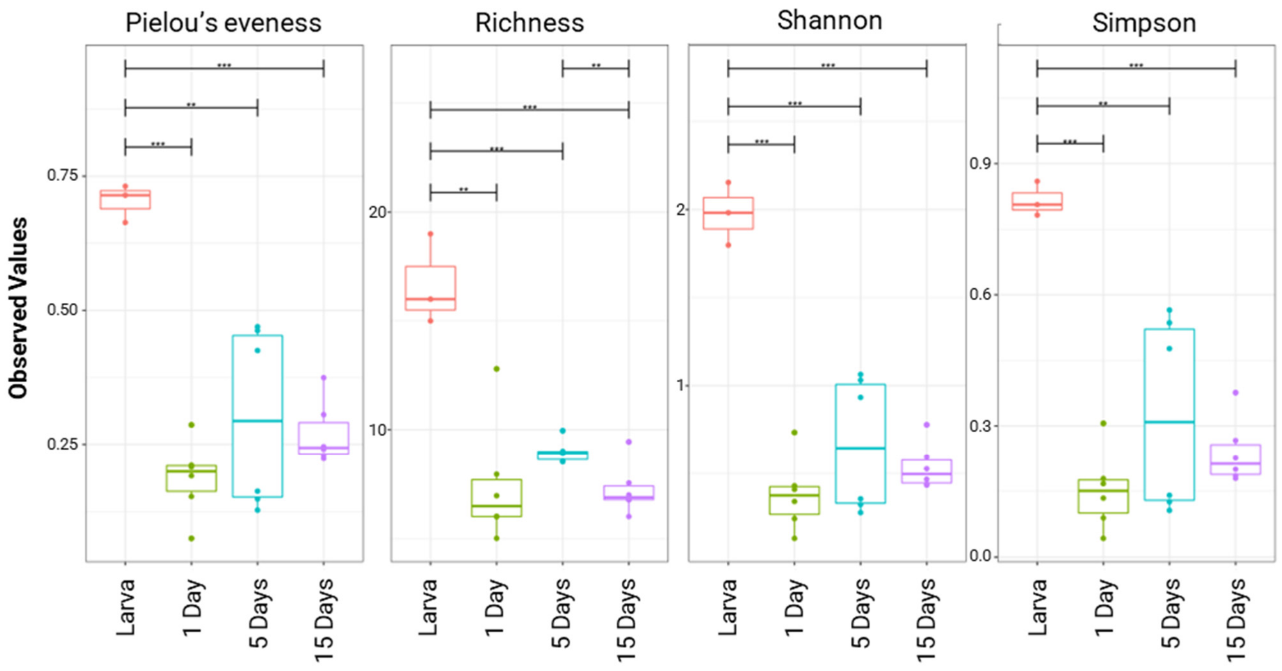

Figure S5: Species richness and diversity indices with significant differences of *G. morsitans* samples according to the age. Boxes represent inter-quartile range (IQR), the line within the boxes is the median, and the dots represent samples. * $p < 0.05$, ** $p < 0.01$ and *** $p < 0.001$.

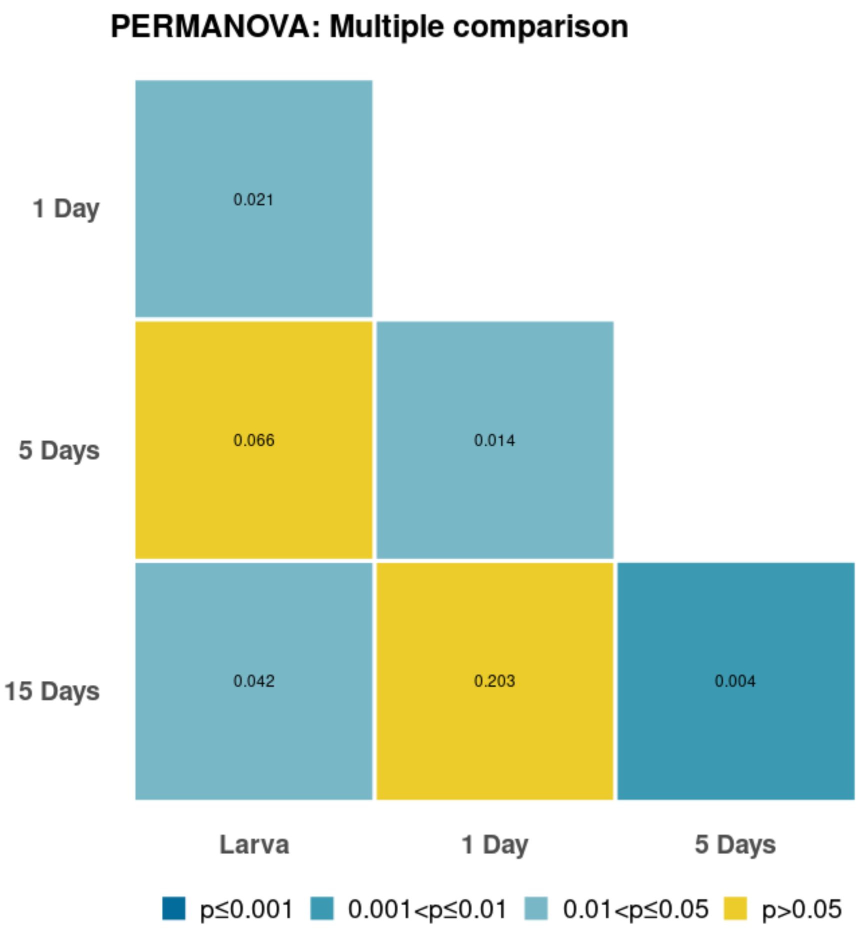

Figure S6: Summary of beta diversity results (pairwise comparison) of the laboratory samples of *G. pallidipes*

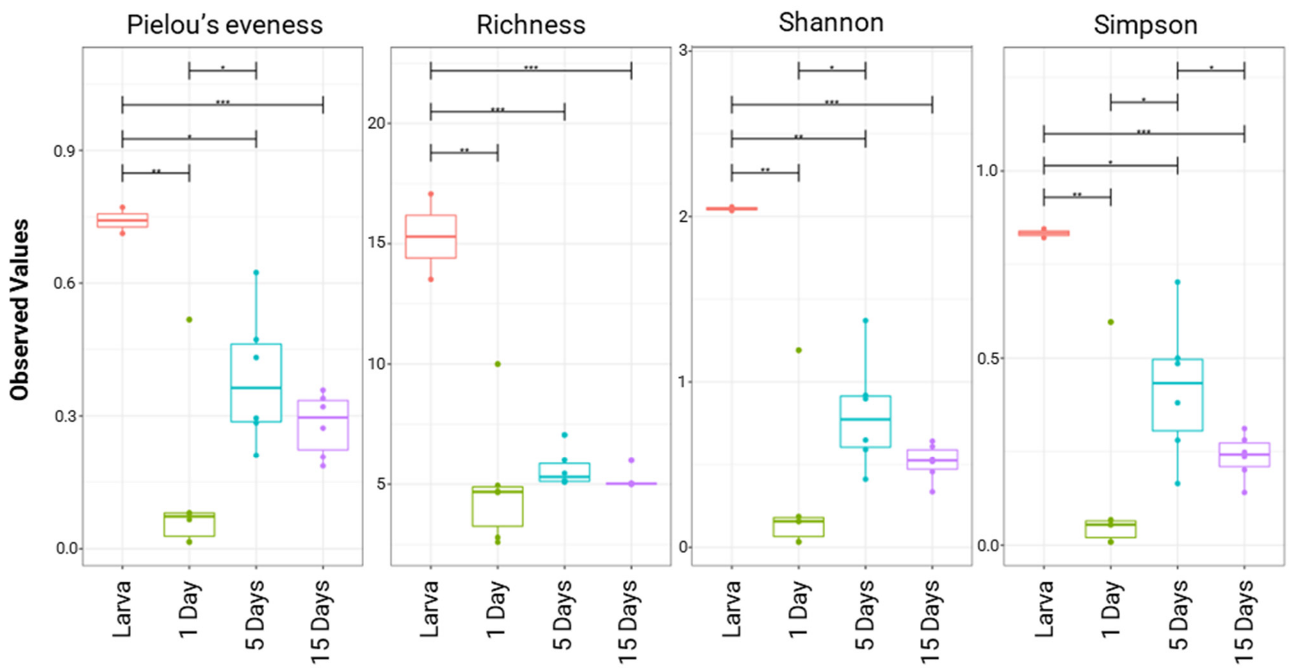

Figure S7: Species richness and diversity indices with significant differences of *G. pallidipes* samples according to the age. Boxes represent inter-quartile range (IQR), the line within the boxes is the median, and the dots represent samples. * $p < 0.05$, ** $p < 0.01$ and *** $p < 0.001$.

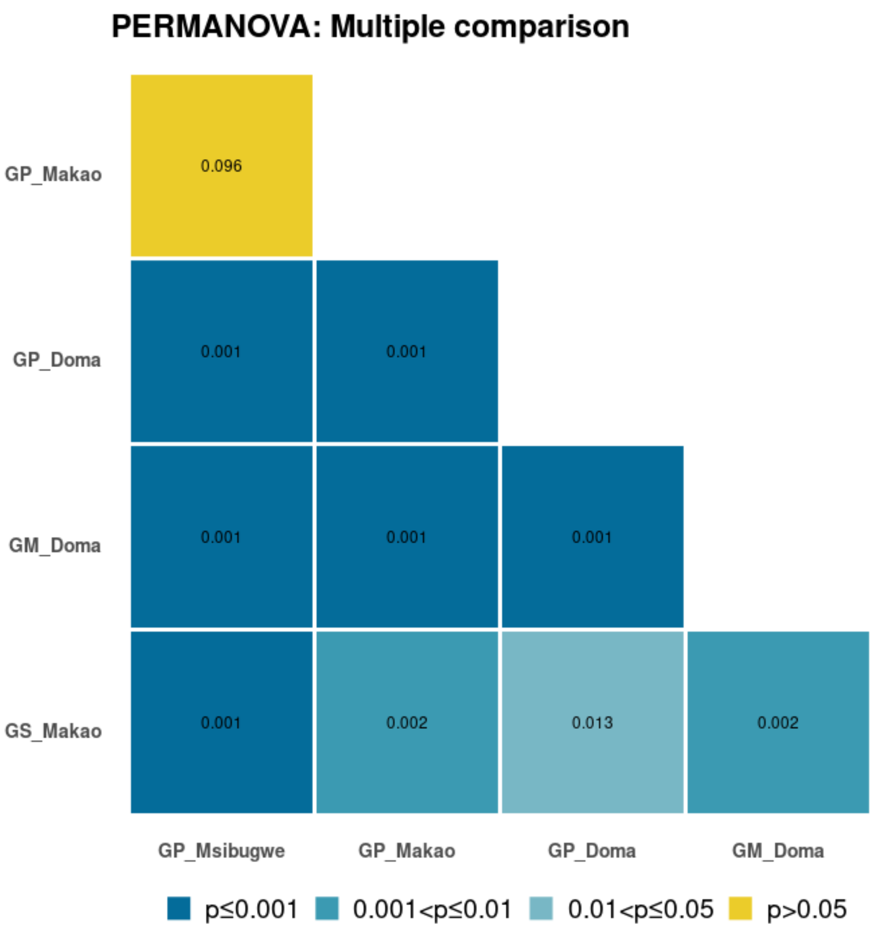

Figure S8: Summary of the beta diversity results (pairwise comparison) of the natural samples.

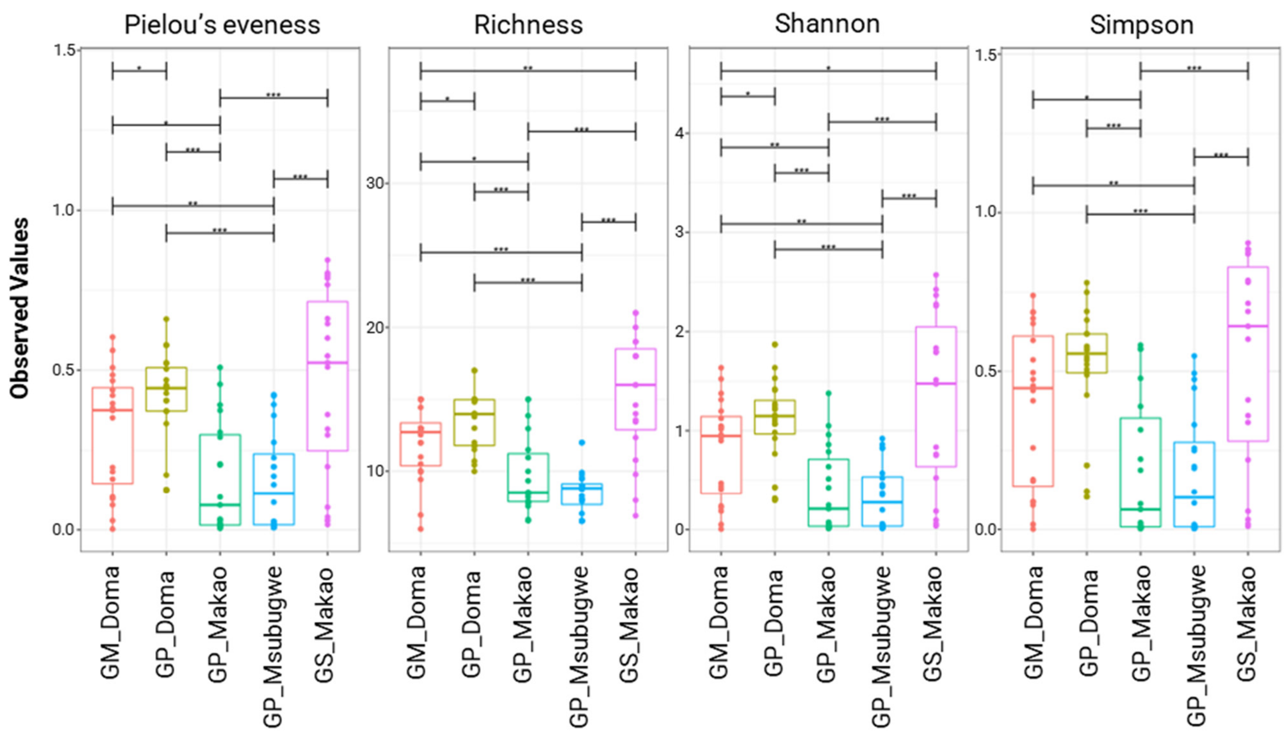

Figure S9: Species richness and diversity indices with significant differences of the natural samples according to the location and the species. Boxes represent inter-quartile range (IQR), the line within the boxes is the median, and the dots represent samples. * p < 0.05, ** p < 0.01 and *** p < 0.001.

Table S3: The percentage of each OTU identified as *Wigglesworthia* or *Sodalis* within the different developmental stages of the laboratory population (GA: *G. austeni*, GM: *G. morsitans morsitans*, GP: *G. pallidipes*, 1D: 1-day-old adults, 5D: 5-day-old adults, 15D: 15-day-old adults). Each percentage derived from the ratio of the number of read counts for each OTU to the total number of reads for each genus, *Wigglesworthia* or *Sodalis*.

		Larva	1D	5D	15D	
Feature ID	Species	% read counts of otu/ total genus reads	% read counts of otu/ total genus reads	% read counts of otu/ total genus reads	% read counts of otu/ total genus reads	Genus
Otu1	GA	59.04	1.36	4.03	1.29	*Wigglesworthia*
Otu3	GA	21.51	0.00	0.00	0.00	*Wigglesworthia*
Otu43	GA	2.75	0.06	0.58	0.16	*Wigglesworthia*
Otu58	GA	14.21	98.10	88.17	97.67	*Wigglesworthia*
Otu84	GA	2.49	0.48	7.22	0.87	*Wigglesworthia*
Otu1	GM	49.72	0.72	0.47	0.22	*Wigglesworthia*
Otu3	GM	28.43	97.83	94.92	95.78	*Wigglesworthia*
Otu43	GM	2.21	1.38	4.44	3.84	*Wigglesworthia*
Otu58	GM	17.21	0.00	0.00	0.00	*Wigglesworthia*
Otu84	GM	2.42	0.07	0.18	0.16	*Wigglesworthia*
Otu1	GP	43.44	99.64	95.83	96.79	*Wigglesworthia*
Otu3	GP	30.68	0.00	0.00	0.00	*Wigglesworthia*
Otu43	GP	2.65	0.21	2.35	1.86	*Wigglesworthia*
Otu58	GP	20.97	0.00	0.00	0.00	*Wigglesworthia*
Otu84	GP	2.26	0.15	1.82	1.34	*Wigglesworthia*
Otu2	GA	85.91	63.36	79.39	61.70	*Sodalis*
Otu50	GA	14.09	36.64	20.61	38.30	*Sodalis*
Otu2	GM	81.69	57.00	73.57	54.98	*Sodalis*
Otu50	GM	18.31	43.00	26.43	45.02	*Sodalis*
Otu2	GP	86.72	90.36	90.01	87.71	*Sodalis*
Otu50	GP	13.28	9.64	9.99	12.29	*Sodalis*
